# Supplementary material for: Comprehensive Annotation and Functional Exploration of MicroRNAs in Lettuce
Source: Front Plant Sci. 2021 Dec 24;12:781836. doi: 10.3389/fpls.2021.781836 (PMC8739914; doi:10.3389/fpls.2021.781836)
Supplement: Supplementary Data 1 — Results of categories 0–2 from PARE-Seq analysis (including three subfiles:1_1, 1_2, 1_3). [file Data_Sheet_10.ZIP › GSM2230755.plot/Lsa-miRN1696_Lsat_1_v5_gn_7_40700.1_259_TPlot.pdf]

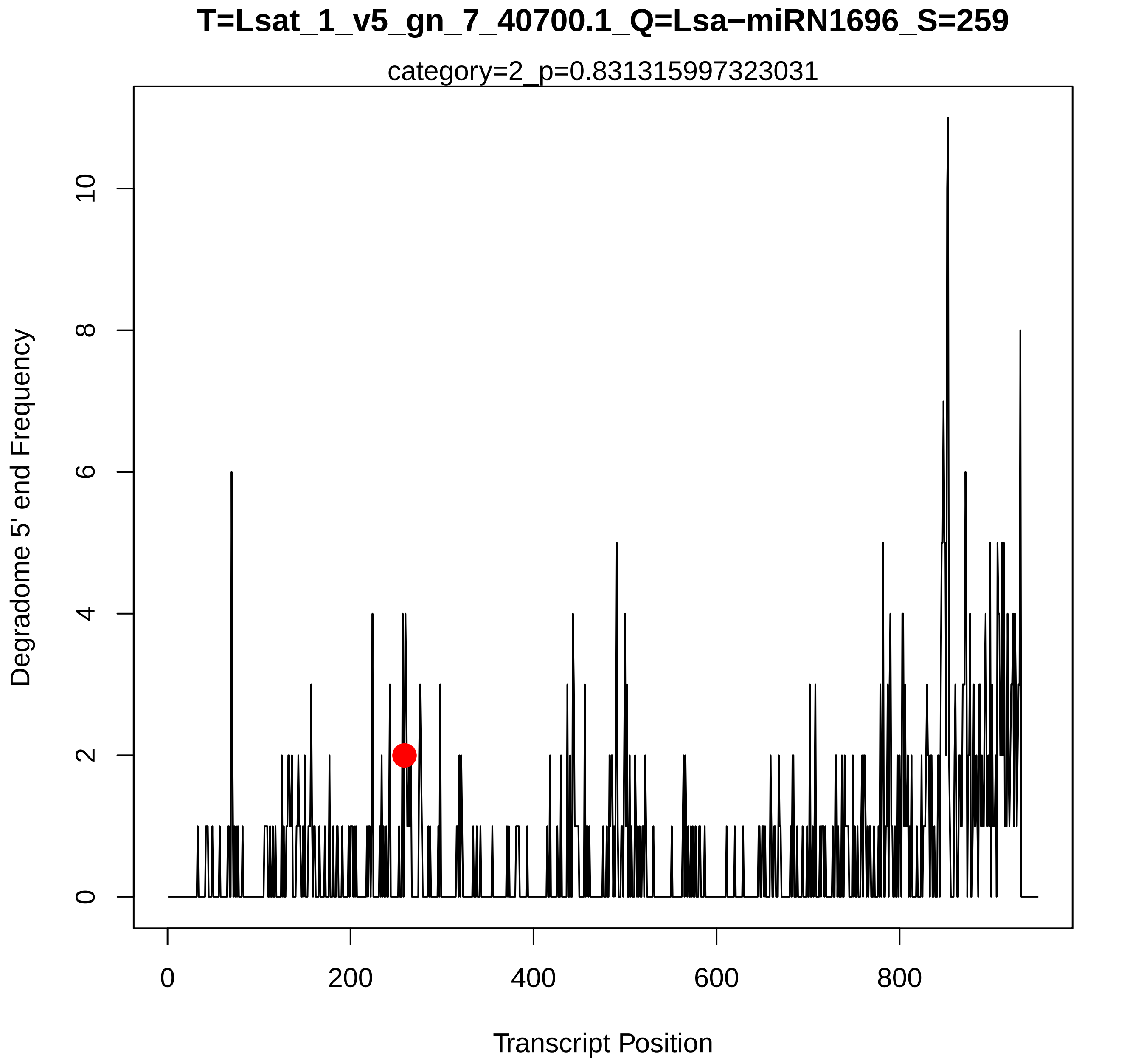
T=Lsat_1_v5_gn_7_40700.1_Q=Lsa−miRN1696_S=259
category=2_p=0.831315997323031
Degradome 5' end Frequency
Transcript Position
0
2
4
6
8
10
0
200
400
600
800
